# A Quantitative Approach to Evaluate the Impact of Fluorescent Labeling on Membrane-Bound HIV-Gag Assembly by Titration of Unlabeled Proteins

**DOI:** 10.1371/journal.pone.0115095

**Published:** 2014-12-10

**Authors:** Julia Gunzenhäuser, Romain Wyss, Suliana Manley

**Affiliations:** 1 Laboratory of Experimental Biophysics, Ecole Polytechnique Fédérale de Lausanne, Lausanne, Switzerland; 2 Laboratory of Physical Chemistry of Polymers and Membranes, Ecole Polytechnique Fédérale de Lausanne, Lausanne, Switzerland; University of Alabama at Birmingham, United States of America

## Abstract

The assembly process of the human immunodeficiency virus 1 (HIV-1) is driven by the viral polyprotein Gag. Fluorescence imaging of Gag protein fusions is widely performed and has revealed important information on viral assembly. Gag fusion proteins are commonly co-transfected with an unlabeled form of Gag to prevent labeling artifacts such as morphological defects and decreased infectivity. Although viral assembly is widely studied on individual cells, the efficiency of the co-transfection rescue has never been tested at the single cell level. Here, we first develop a methodology to quantify levels of unlabeled to labeled Gag in single cells using a fluorescent reporter protein for unlabeled Gag and fluorescence correlation spectroscopy. Using super-resolution imaging based on photoactivated localization microscopy (PALM) combined with molecular counting we then study the nanoscale morphology of Gag clusters as a function of unlabeled to labeled Gag ratios in single cells. We show that for a given co-transfection ratio, individual cells express a wide range of protein ratios, necessitating a quantitative read-out for the expression of unlabeled Gag. Further, we show that monomerically labeled Gag assembles into membrane-bound clusters that are morphologically indistinguishable from mixtures of unlabeled and labeled Gag.

## Introduction

Viral infection of a cell is ultimately marked by the assembly and release of progeny viral particles. In the case of retroviruses such as HIV-1 this process is driven by the viral polyprotein Gag. A remarkable feature of Gag is its ability to assemble into virus-like particles (VLPs), even in the absence of any other viral component [1], [2]. Direct observation of viral formation was made possible by the use of fluorescent protein tags (FPs), which permit non-invasive, specific live-cell imaging. Real-time monitoring of the Gag assembly process has recently revealed information on the time scale of viral formation and Gag assembly kinetics [3], [4]. The constant improvement of FPs and fluorescence imaging techniques make them promising tools for elucidating still open questions on HIV assembly such as how Gag proteins reach their assembly sites, or how the interplay between Gag and viral RNA is orchestrated during VLP formation. A concern with the use of FPs is their potential interference with protein function or localization [5]. In the case of Gag the impact of a fluorescent label is controversial, and different studies have reached disparate conclusions on whether and how the FP affects viral assembly and morphology [6]–[9]. Despite these controversies, two distinct plasmids are generally co-transfected for fluorescent Gag studies, one encoding for a Gag-FP constructs and a second encoding for unlabeled Gag, usually in a ratio from 1∶1 to 1∶10 [3], [4], [8]–[15].

However, for the co-transfection of unlabeled Gag to be meaningful for single-cell or single particle studies, it must be homogenous for all analyzed assembling or budded VLPs. Current interpretations assume incorporation of both types of Gag into VLPs, as well as comparable ratios of both forms within each imaged or producer cell. Here, we develop a methodology to quantify expression levels of unlabeled Gag in single cells using a fluorescent reporter protein for unlabeled Gag and fluorescence correlation spectroscopy (FCS) [16]–[18]. We then combine this methodology with super-resolution imaging [19]–[21] and molecular counting [7], to resolve the morphology and to estimate the number of Gag proteins in individual Gag clusters. Using this approach we directly study the nanoscale morphology of membrane-bound forming VLPs as a function of unlabeled to labeled Gag ratios in single cells. This allows us to reveal important differences between bulk and single cell measurements when co-transfection procedures are used.

## Materials and Methods

### Cell culture and transfection

African green monkey kidney cells **(**Cos7) or HeLa cells were purchased from Health Protection Agency Culture Collections (HPA Culture Collections) and cultured in DMEM supplemented with 10% FBS (Sigma Aldrich). For PALM imaging, cells were plated on 25 mm coverslips, cleaned with 1∶1∶5 H_2_O_2_/NH_4_OH/H_2_O for 3 h at 70°C and coated with 100 nm Au beads serving as fiducial markers to remove the effects of sample drift, 48 h prior to imaging. Cells were transfected with 4 µg of the appropriate plasmids in warm 100 µl DMEM without FBS and 6 µl FuGene6 (Roche Diagnostics) incubated for 15 min, 24 h prior imaging. For co-transfection, plasmids were mixed in warm 100 µl DMEM without FBS at indicated ratios (ranging from 1∶1 to 1∶5) to reach a final amount of DNA of 4 µg and incubated together with 6 µl FuGene6 for 15 min. For PALM imaging cells were fixed by incubation with 4% paraformaldehyde in PBS for 15 min just before imaging.

### Plasmids and cloning

pGag-EGFP has been described previously [22]. To produce the Gag-mCherry, a Rev-independent HXB2 Gag sequence was excised from pGagEGFP by digestion with XhoI and BamHI. The resulting fragment was inserted in frame into the XhoI and BamHI sites of the mCherry-N1 plasmid. *pGag-mEos2*, was obtained by replacing BamHI/XbaI fragment of Gag-tdEos [23], with the *BamHI*/*XbaI* fragment of pmEos2-N1 [24]. The *H2B-mPlum* reporter gene was amplified from the *pH2B-mPlum* plasmid (also provided by G. Patterson) using the following primers:

5′-CTAGAGATCTGCCACCTTCTGCAGTCGACGGTACC-3′.

5′-CTAGGCTAGCCCTCTACAAATGTGGTATGGC-3′. The PCR product was digested by *BglII* and *NheI* and introduced into the BamHI/AvrII sites of the mammalian multigenic plasmid pvitro1-nmcs (InvivoGen) which allows ubiquitous and constitutive co-expression of two genes of interest. *Gag* was PCR amplified (from *pGAG-tdEos*) using the following primers: 5′-CTAGGGATCCAAGCAGAGCTGGTTTAGTGAACCG-3′ and 5′-CTAGTCTAGATTGTGACGAGGGGTCGTTGCCAA-3′. The PCR product was digested with BglII and NheI and introduced into *pVITRO-H2B-mPlum* cut with the same enzymes to give the final vector *pH2B-mPlum/Gag.*

### Immunostaining

Cells were prepared as for PALM imaging, washed 3 times 5 min each time in PBS, permeabilized for 10 min in PBS+0.3% Triton X-100 and washed again 3 times, 5 min each time in PBS. Cells were than incubated with HIV-1 p55+ p24+ p17 rabbit polyclonal antibody (Abcam) at a dilution of 1/20000 in PBS+10% FBS+0.3% Triton for 2 h at 37°C, washed 3 times 5 min, each time in PBS. Finally, cells were and incubated with anti-rabbit-Alexa 488 (1/1000, Invitrogen) for 1 h at 37°C, washed again 3 times 5 min, each time in PBS and imaged immediately.

### Western blot

For electrophoretic analysis and protein quantification, total cell lysates (fast break buffer, Promega) from approximately 5×10^5^ cells (transfected with *pH2B-mPlum/Gag*, in triplicate), recombinant HIV-Gag (B-Bridge International) and mPlum (Proteogenix) protein standards were boiled for 5 min in loading buffer (62.5 mM Tris/Cl, 2% SDS, 10% glycerol, 1 mM DTT and 0.25% bromophenol blue). We note that both protein standards were produced from bacteria and are not myristylated. Thus, the efficiency of transfer is not guaranteed to be equal for protein standards and cell lysates. Samples were separated on a 12% SDS-PAGE at 150 V for 90 min, electro-blotted at 100 V for 60 min on a PVDF membrane and blocked with TBS/T (25 mM Tris, 150 mM NaCl, 2 mM KCl, 0.1% Tween 20, pH 7.4) containing 5% non-fat dry milk (Biorad). For immunodetection, PVDF membranes were sequentially incubated 90 min at 4°C with specific antibodies directed against RFP (1:500, Ab28664, Abcam) first and then antibodies against HIV-Gag (1:2000, Ab63917, Abcam). For each staining the membrane was washed three times with TBS/T and incubated for 30 min with the secondary antibody (1:2500 HRP conjugated goat anti-rabbit IgG, Amersham). The immunoreactive bands were detected with enhanced chemiluminescence ECL2 Blotting Substrate kit (Pierce) and revealed on a Fujifilm LAS4000 biomolecular imager (GE Healthcare). Quantification of the immunoblots was performed using Fiji.

### Fluorescence correlation spectroscopy

FCS measurements were performed with a LSM 510 Meta laser scanning microscope based on an Axiovert 200 M stand and equipped with a ConfoCor 3 unit for FCS (Zeiss). The setup allowed acquisition of photon-time traces and online correlation of the data. mEos and mPlum fluorescent proteins were excited at 488 nm from the built-in Ar-ion laser (LGK7812 ML4, Lasos Lasertechnik GmbH) and at 561 nm from the built-in diode-pumped solid-state laser (85-YCA-010, Melles Griot), respectively. An acousto-optical filter (AOTF) was used to adjust the incident irradiance after the microscope objective (40x C-Apochromat, NA 1.2, water immersion, Zeiss) below 2 kW/cm^2^ for Ar-ion laser and 33 kW/cm^2^ for YLK 6110 T laser to reduce photobleaching and photophysical effects. Systematic determination of the lateral beam waist radius ω_0_ of the focused Ar-ion and 85-YCA-010 lasers was performed by measuring the translational diffusion time constants τ_D_ of soluble Alexa Fluor 488 (Invitrogen) and Cy3 (GE Healthcare), respectively, with known diffusion coefficients D in aqueous solution according to ω_0_ = (4Dτ_D_)^1/2^. Protein concentration determination inside cells was performed in two steps. First, fluorescence intensity time traces and correlation curves of 50 µl drops containing various concentrations of either purified mEos2 (kind gift from Prof. Radenovic, EPFL) or mPlum (Proteogenix) in aqueous solution supplemented with 1% BSA were recorded for 10×10 s on a coverglass. The autocorrelation data was fitted within Zeiss AIM software (Zeiss) using the equation:
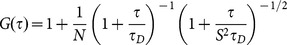
where τ is the lag time, N is the total number of fluorescent protein in the observation volume, τ_D_ is the translational diffusion time constant of the protein and S is the structure parameter defined by the ratio of the axial and lateral axes of the observation volume. For this first calibration step we used molar concentrations ranging from 1 to 300 nM, the optimal range for FCS measurements (S3 and S4 Figures in S1 File). The calibration gave a linear relation of the actual concentration (measured by absorption) vs. the concentration measured by FCS with a slope off 1.7 for mPlum and 4.8 for mEos2 (S5 Figure in S1 File). This approach allows us to account for non-fluorescent or photoactivated (mEos2) molecules and explains the high nM concentrations obtained for the final calibration curve. Second, fluorescence intensity time traces and correlation curves of cells cultured on a coverglass as for LM imaging and expressing either cytosolic mEos2 or mPlum were recorded for 10×10 s (S3 and S4 Figures in S1 File). We assume that for mPlum the relevant photophysical parameters such as brightness and quantum yield are similar in the cytoplasm and nucleus. Cells with high expression levels or presenting any fluorescence heterogeneity were discarded. For each cell selected, 2 to 3 different positions of the observation volume were chosen near the center of the cell, but within the cytosol. Each FCS run was examined, and fluorescent traces presenting multi-exponential decays or significant slow components (corresponding to larger protein complexes or membrane-bound proteins) were not taken into account. The FCS data were best fit to single or double exponential decays (S3 and S4 Figures in S1 File). However, the determined cellular concentration of fluorescent protein was not significantly affected by the number of components used for fitting, since the amplitude and not the decay time is used to determine the concentration. The integrated fluorescence intensity normalized by the surface of the fluorescence distribution of each cell was extracted using Fiji and plotted against its concentration (taking into account the first calibration curve). The extrapolated linear fit of this data provided the function to convert from the normalized integrated fluorescence intensity to fluorescent protein concentration.

### Super-resolution imaging

Cells were imaged using a Zeiss Axio Observer D1 inverted microscope, equipped with a 100x, 1.49 NA objective (Zeiss). Activation and excitation lasers with wavelengths 405 nm (Coherent cube) and 561 nm (Crystal laser) illuminated the sample in total internal fluorescence (TIRF) mode. We used a four color dichroic 89100bs (Chroma), fluorescence emission was filtered with an emission filter ET605/70 (Chroma) and detected with an electron-multiplying CCD camera (iXon+, Andor Technology) with a resulting pixel size of 160 nm. For each region of interest, typically 10000 images of a 41×41 µm^2^ area were collected with an exposure time of 30 ms. The irreversible photoactivatable protein mEos2 was activated with continuous 405 nm laser intensity of <0.5 W/cm^2^, to guarantee very sparse, spatially well separated activation of molecules and minimize blinking, and excited with 561 nm laser intensity of ∼1 kW/cm^2^. Molecules were localized and super-resolution images rendered using Peakselector (IDL, courtesy of Harald Hess). The single molecule localization procedure consisted of the following steps: a) fluorescent intensity peaks were detected on each image, b) each peak was fitted to a two-dimensional Gaussian by nonlinear least-square fitting to obtain x and y coordinates as well as fitting parameters, c) images were dedrifted using Au fiducial markers, d) localizations detected within less than the measured mean localization precision (typically between 19 and 24 nm) in space and 300 ms in time were grouped to account for blinking of mEos2. One grouped molecular position is counted as one Gag-mEos2 protein [7]. Only molecules localized to better than 30 nm were used for analysis and rendering. Grouped molecular positions were rendered as Gaussians, with color corresponding to the probability density. Overlapping Gaussians were rendered showing the resulting envelope to emphasize molecular positions. The determined number of molecules was used to compute the VLP density (see next section).

### Morphology and density analysis of membrane-bound Gag clusters

Clusters were extracted as described [7]. In brief, clusters containing a minimum number of 32 peaks were identified based on an adapted Hoshen–Kopelman algorithm. We chose a maximum distance between molecules belonging to the same cluster of 50 nm, a distance at least 5 times less than mean inter-molecular distances within non-clustered regions. For each cluster the center of mass (CM) is computed. The CM serves as origin to define a grid that divides each π/2 radians into 4 equally sized sectors. To parameterize the shape and size of each cluster, the mean radius of each sector is extracted. The radius of each cluster is computed as the average of the mean radius over the total 16 sectors. The radial anisotropy is defined as the standard deviation of the average mean radius divided by the mean radius. The density of each cluster is computed by dividing the number of identified molecules by the area covered by the VLP.

## Results

### Construction of a fluorescent read-out for the expression of unlabeled Gag

VLP formation is generally studied by co-transfecting plasmids encoding for labeled and unlabeled Gag. When two fluorescently labeled proteins are co-transfected, such as Gag-GFP and Gag-mCherry, visual selection for co-expressing cells can be performed (S1 Figure in S1 File). For this pair of fluorescently labeled Gag under identical promoters we found that 100% of Gag-mCherry expressing cells also expressed Gag-GFP. However, only 14% of Gag-GFP positive cells also expressed Gag-mCherry (two independent transfections, number of cells: >400). As a consequence, the pre-selection of co-expressing cells for imaging and analysis is necessary since transient co-transfection does not guarantee co-expression at the single-cell level [25], [26]. In the case of Gag, to rescue native infectivity, one of the transfected proteins must be unlabeled, precluding any fluorescence-based direct selection for co-expressing cells.

**Figure 1:**
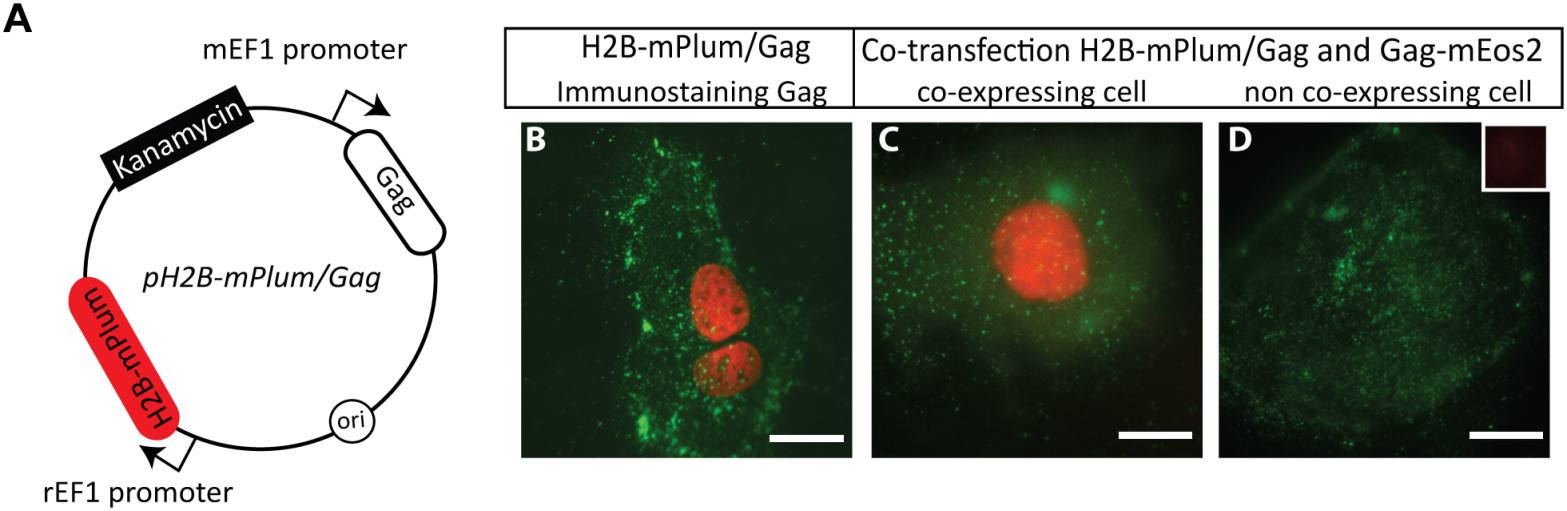
Visualization of unlabeled Gag expression in single cells. (*A*) Plasmid map of *pH2B-mPlum/Gag* (*B*) Wide-field images of Cos7 cells transfected with the *pH2B-mPlum/Gag* vector and immunostained against Gag (Alexa Fluor 488). The nuclear H2B-mPlum reporter protein (red) and Gag (green) are always present in conjunction. (*C*) Cells co-transfected with a mixture of *pH2B-mPlum/Gag* and *pGag-mEos2* at an equimolar ratio. Image of H2B-mPlum (red) and Gag-mEos2 (green) of a typical cell expressing both constructs. (*D*) Cells co-transfected with a mixture of *pH2B-mPlum/Gag* and *pGag-mEos2*, image of a cell expressing only Gag-mEos2. The inset shows the fluorescence detected in the red channel, which is at the background level. Scale bars correspond to 5 µm.

An alternative to co-transfection of two separate plasmids is to encode both proteins of interest on a single plasmid. However, this does not permit straightforward titration of the levels of each protein. Indeed, for the commonly used ratio of 1∶10 labeled to unlabeled Gag plasmid, there is no obvious and robust strategy to bypass this co-transfection. To address this issue and identify cells that are expressing the unlabeled form of Gag we used a fluorescent reporter protein incorporated into the same plasmid. We chose the fluorescently labeled histone H2B-mPlum as the fluorescent reporter because its nuclear localization minimizes interference with the fluorescent signal of Gag in the cytosol and plasma membrane. We constructed a single plasmid encoding at the same time for the two genes of interest, H2B-mPlum and Gag, under similar promoters, *pH2B-mPlum/Gag* as a proxy (Fig. 1*A*; Materials and Methods).

Cells transfected with *pH2B-mPlum/Gag* display a nuclear staining, as is expected for histones (Fig. 1, *B* and *C*). The simultaneous expression of unlabeled Gag was confirmed by immunostaining (Fig. 1*B*). Thus, using this construct cells expressing the unlabeled form of Gag can be identified by their red/far-red nuclear stain. For the labeled form of Gag, we fused Gag to mEos2 the monomeric form of the green-to-red irreversibly photoconvertable FP Eos [24]. Co-transfecting the *pH2B-mPlum/Gag* construct together with Gag-mEos2 at an equimolar ratio revealed that only 43% of transfected cells were expressing both constructs (two independent transfections, number of cells = 200). This again highlights that for experiments at the single cell level, a read-out for the expression of unlabeled Gag is required to select cells expressing both forms of Gag.

### The created fluorescent reporter provides a quantitative read-out for the expression of unlabeled Gag

**Figure 2:**
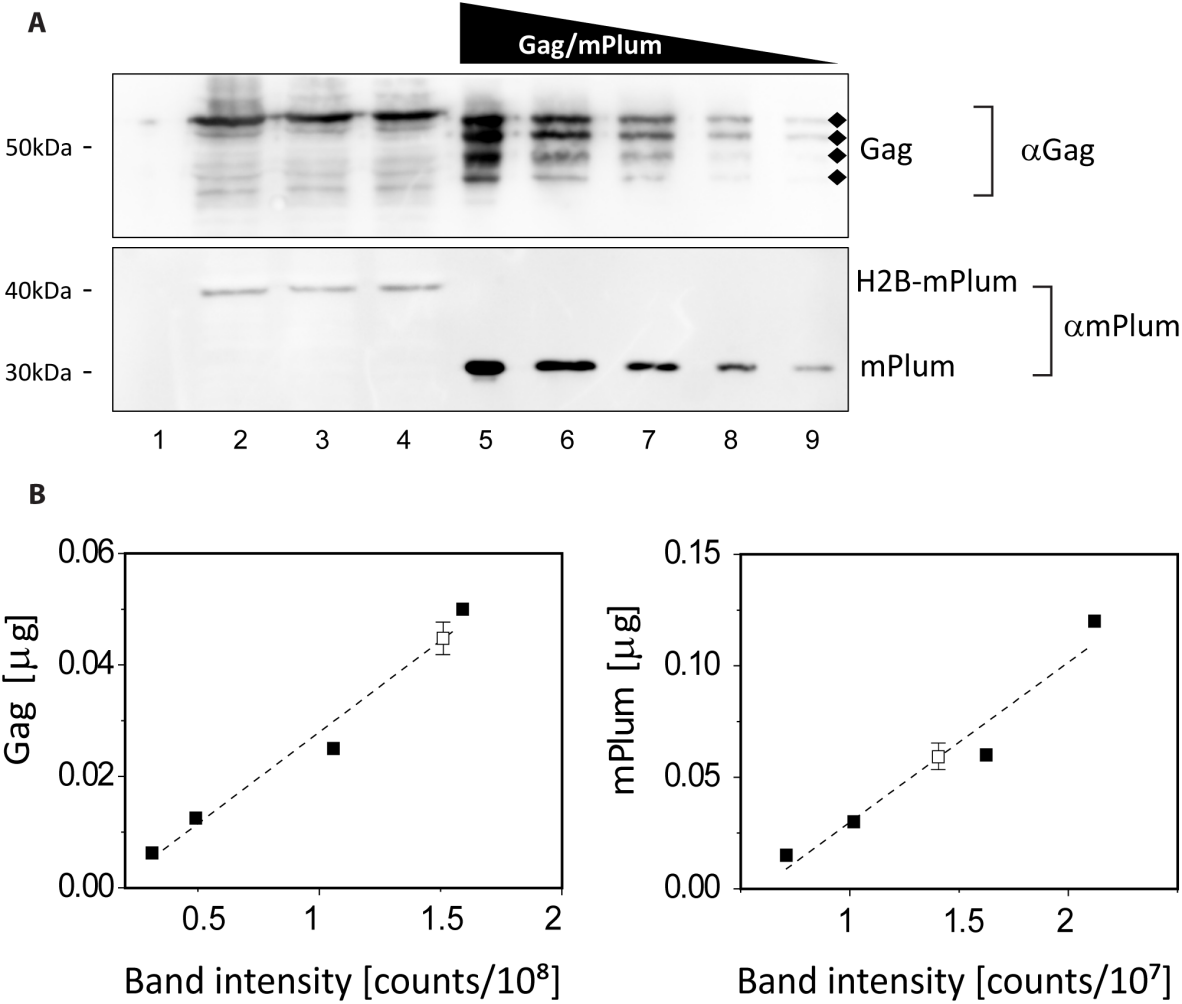
Quantifying the bulk expression levels of H2B-mPlum and Gag. (*A*) Quantitative western blot. Lane 1: non-transfected cells. Lanes 2, 3, 4: biological replicates of total cell extracts from Cos7 cells transfected with the *pH2B-mPlum/Gag* construct. Gag (the diamonds indicate bands corresponding to degradation products that were included in the quantification) and H2B-mPlum were detected using specific anti-Gag and anti-RFP antibodies. Lanes 5, 6, 7, 8, 9: purified Gag (upper panel) and mPlum (lower panel) ranging from 6.25 to 100 ng and 15 to 240 ng respectively. (*B*) Calibration curves obtained from serial protein dilutions (closed squares, from lanes 5–9) and measured Gag and H2B-mPlum concentration in cell extracts (open square, from lanes 2–4, mean and standard deviation for the triplicate are shown). The bands corresponding to the highest concentrations were omitted in the fit, their intensity being close to saturation.

The presence of the fluorescent reporter H2B-mPlum provides only a qualitative read-out of unlabeled Gag in single cells (Fig. 1, *B–D*). If we can quantify the relative expression levels of the reporter protein and Gag, encoded from similar promoters within the same plasmid, it could also provide a quantitative read-out of unlabeled Gag expression. So, we assessed the total expression of H2B-mPlum and Gag by quantitative western blotting (Fig. 2, *A* and *B*).

Cos7 cells transfected with *pH2B-mPlum/Gag* were lysed and proteins were isolated by sodium dodecyl sulfate - polyacrylamide gel electrophoresis (SDS-PAGE). Proteins were western-blotted and revealed by immunostaining with antibodies directed against Gag and mPlum, to omit signal from the endogenous population of H2B (Fig. 2*A*, lanes 2–4 upper panel). In order to quantify the levels of Gag and H2B-mPlum expressed in cells, a range of purified Gag (100 to 6.25 ng) and mPlum (240 to 15 ng) was loaded on the same gel (Fig. 2*A*, lanes 5–9). The calibration curves obtained from the purified protein dilutions (Fig. 2*B*) were used to determine bulk cellular expression levels of Gag and H2B-mPlum [27]. Using this quantification approach we inherently correct for labeling efficiencies of the different primary antibodies against mPlum and Gag. The western blotting of three independent cell-extracts confirmed that transfection of *pH2BPlum/Gag* yields similar bulk expression levels of Gag and H2B-mPlum with a mass ratio of 0.8±0.1 (mean ± SD.). We also verified that this holds at the single cell level (S2 Figure in S1 File). Thus, the amount of unlabeled Gag expressed upon transfection with *pH2B-mPlum/Gag* can be measured indirectly but quantitatively by measuring the amount of the fluorescent reporter H2B-mPlum.

### Correlating single cell fluorescence intensity with protein concentration via FCS

**Figure 3:**
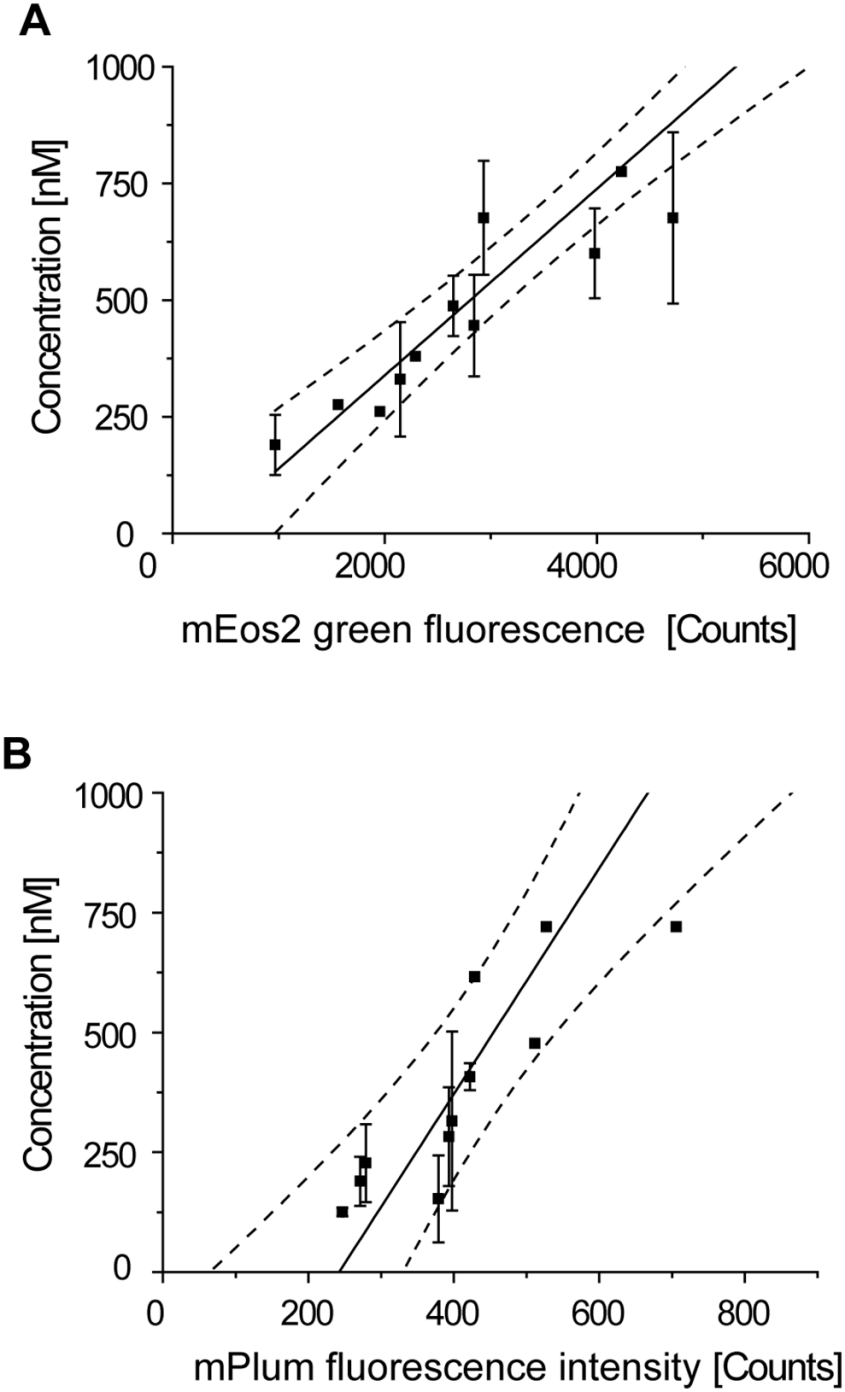
Calibration curves obtained with FCS. The molar concentration of (*A*) mEos2 and (*B*) mPlum is shown as a function of the integrated cytosolic fluorescence intensity. Counts are given in arbitrary units and refer to the integrated fluorescence intensity of the wide-field image of the cell. Mean and standard deviation from three measurements within one cell are shown. A linear fit (solid line) with a 95% confidence interval (dashed lines) is shown. The extrapolated linear fit of the fluorophore concentration as a function of the integrated fluorescence intensity was used for the determination of protein expression levels.

The western blot approach allowed us to quantify bulk protein concentrations. VLP assembly however, is studied at the single cell level. For single cells the concentration of FPs is reflected by the total integrated fluorescence intensity of the whole cell (for Gag-mEos2) or the nucleus (for unlabeled Gag via the reporter H2B-mPlum). The FPs mPlum and mEos2 used in this study thus provide *per se* read-outs for single cell expression levels of Gag and Gag-mEos2. The quantitative relationship between fluorescence intensity and protein concentration, however, also depends on the brightness of each FP. To account for this, we used FCS, which permits the experimental determination of molar concentration of FPs in living cells, and in turn can be related to the measured fluorescence intensity. FCS measures spontaneous fluorescence intensity fluctuations in the femtoliter focal volume. The temporal correlation of these fluctuations reflect protein concentrations and mobilities. We performed FCS in living cells to obtain calibration curves relating the protein concentration of mEos2 (Fig. 3*A*) and mPlum (Fig. 3*B*) to the mean cytosolic fluorescence intensity (S3 and S4 Figures in S1 File).

This calibration was linear as expected and gave a ratio of mEos2 (green form) to mPlum brightness of 12. This is in very good agreement with the ratio 11.5 determined from published values [24], [28]. We note that the integrated fluorescence intensity is assessed from two-dimensional wide-field images (Fig. 1), which contributes to the uncertainty in the estimation of both protein concentrations since they are distributed in three dimensions. In contrast to the information on bulk expression levels obtained by western blotting, the calibration curves obtained by FCS permit the determination of protein expression levels for single cells. We will use this quantification of protein expression at the single cell level to study cell-to-cell variances in the co-expression of labeled and unlabeled Gag.

### Protein co-expression ratios reveal high variability at the single cell level

**Figure 4:**
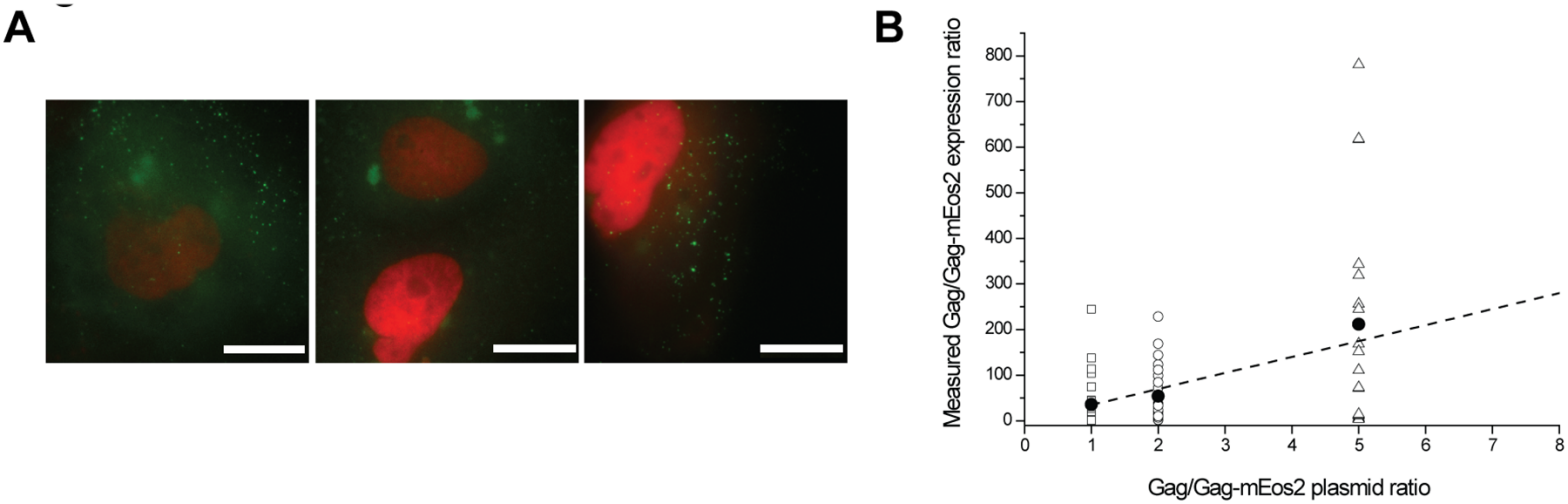
Variability of protein co-expression ratios at the single cell level for identical co-transfection conditions. (*A*) Wide-field images of cells co-transfected with a mixture of *pH2B-mPlum/Gag* and *pGag-mEos2* at an equimolar ratio show high variability in H2B-mPlum expression. (*B*) Measured Gag/Gag-mEos2 co-expression ratios as a function of the co-transfection ratio of plasmids. Each symbol corresponds to the co-expression ratio measured in a single cell. The black circles show the mean values. The linear fit of the mean co-expression level is plotted as a dashed line. The expression levels were extracted from the extrapolated FCS calibration curves shown in Fig. 3. Scale bars correspond to 5 µm.

Previous studies have shown that the unlabeled to labeled Gag stoichiometry scales linearly with plasmid stoichiometry upon co-transfection as measured by bulk assays, performed on samples consisting of hundreds of thousands of VLPs produced by thousands of cells [6]. However, the critical biological process of Gag assembly and VLP formation in live cells is studied at the single cell level. Single cell measurements of protein stoichiometry are thus necessary to assess whether linear scaling is also preserved at the single cell level. Using our developed quantification tools, we studied the correlation between the co-transfection and co-expression ratio of Gag/Gag-mEos2 at the single cell level. We co-transfected cells with different plasmid stoichiometries ranging from equimolar concentrations of *pGag-mEos2* and *pH2B-mPlum/Gag* to a fivefold excess in p*H2B-mPlum/Gag* and measured protein stoichiometry (Fig. 4) using the calibration curves obtained with FCS. We note that the commonly used tenfold excess in unlabeled Gag could not be used here because in those conditions Gag-mEos2 expression was too low for unambiguous identification and quantification of Gag-mEos2 levels in single cells.

We found the mean Gag/Gag-mEos2 expression ratio (N>18, for each plasmid stoichiometry) to be linearly related to the co-transfection ratio, confirming previous bulk biochemical studies [6] (Fig. 4*B*). Interestingly, there was a high variability between plasmid and protein stoichiometry at the single cell level (Fig. 4*A*). Cell-to-cell differences in expression levels led to a wide range of stoichiometries between labeled and unlabeled Gag, for all transfection conditions studied. These observations indicate that the plasmid stoichiometry used for transfection is not robustly reflected at the single cell level, emphasizing the need for quantitative read-outs of helper Gag expression levels in single cell studies.

### Labeled and unlabeled Gag proteins are incorporated into forming VLPs

**Figure 5:**
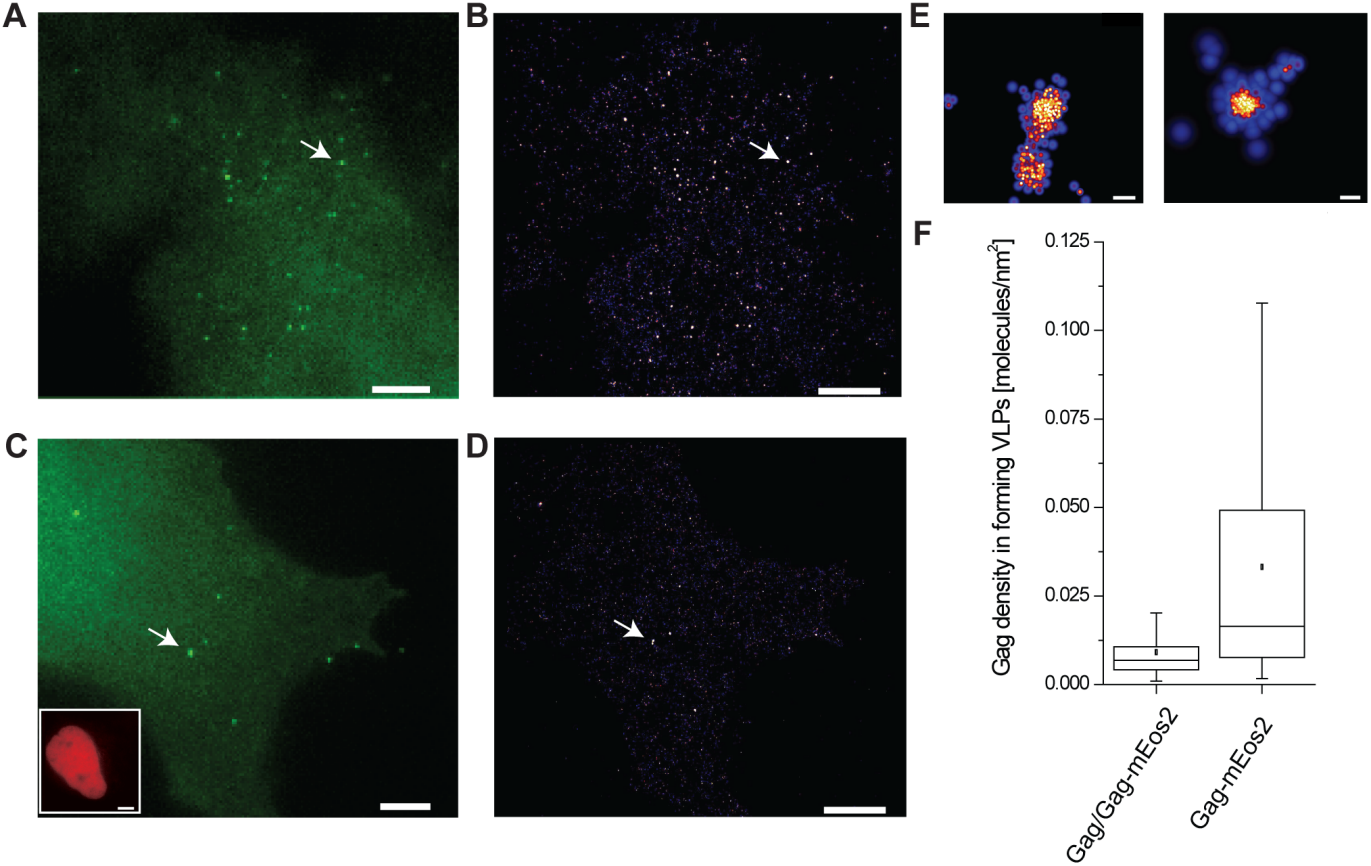
Unlabeled and labeled Gag protein incorporation into forming VLPs. (*A*) Diffraction limited image and (*B*) corresponding super-resolution image of a Cos7 cell expressing Gag-mEos2 only. (*C*) Diffraction limited image and (*D*) corresponding super-resolution image of a Cos7 cell co-expressing Gag and Gag-mEos2, co-transfected at an equimolar ratio. The presence of Gag is indicated via the fluorescent reporter protein H2B-mPlum (lower left inset). Scale bars, 5 µm. (*E*) Left, zoom of indicated VLP in (B). Right, zoom of indicated VLP in (D). Scale bars, 100 nm. (*F*) Gag density in forming VLPs as determined from PALM images and molecular counting. Closed squares indicate the mean, horizontal lines within the boxes median, and boxes indicate 25^th^ and 75^th^ percentiles while vertical bars indicate 5^th^ and 95^th^ percentiles. Cells co-expressing unlabeled and labeled Gag display lower densities, indicating the presence of unlabeled Gag proteins within clusters, invisible to fluorescence detection.

We used our quantitative read-outs for single-cell expression levels to identify and examine cells effectively expressing relative concentrations of fluorescently labeled to unlabeled Gag over a range of an order of magnitude (Fig. 4*B*). Changing Gag protein stoichiometry in a cell does not *a priori* guarantee an incorporation of both types of Gag into VLPs. Thus, we tested whether unlabeled Gag is incorporated by quantitative PALM imaging and molecular counting analysis. Cells expressing Gag-mEos2 alone (Fig. 5, *A* and *B*) or Gag and Gag-mEos2 (Fig. 5, *C* and *D*) were first imaged in wide-field in the green channel to record diffraction-limited images of forming VLPs (Fig. 5*A* and *C*) and in the red/far-red channel to detect and quantify the expression level of H2B-mPlum and thus unlabeled Gag (Fig. 5*C*, inset). We then acquired data for PALM images by stochastically photoconverting Gag-mEos2 molecules into the red form with 405 laser light. Individual molecules were imaged, bleached, subsequently localized and their positions were rendered to form a composite PALM image (Fig. 5, *B* and *D*). Within PALM images we can resolve single clusters of Gag-mEos2 (Fig. 5*E*). We can moreover estimate the number and density of labeled proteins in nascent VLPs taking into account the photo-physical properties of mEos2 such as the average blinking rate and dark-time [7]. This analysis confirmed that the density of labeled Gag proteins within Gag clusters decreased when unlabeled Gag was co-expressed (Fig. 5*F*). This indicates incorporation of both labeled and unlabeled Gag proteins into forming VLPs, confirming previous work [9]. However, we did not find a clear correlation between the amount of unlabeled Gag within a single cell and the number of labeled Gag proteins per cluster.

### Labeled and unlabeled/labeled mixtures of Gag form morphologically indistinguishable clusters at the membrane

**Figure 6:**
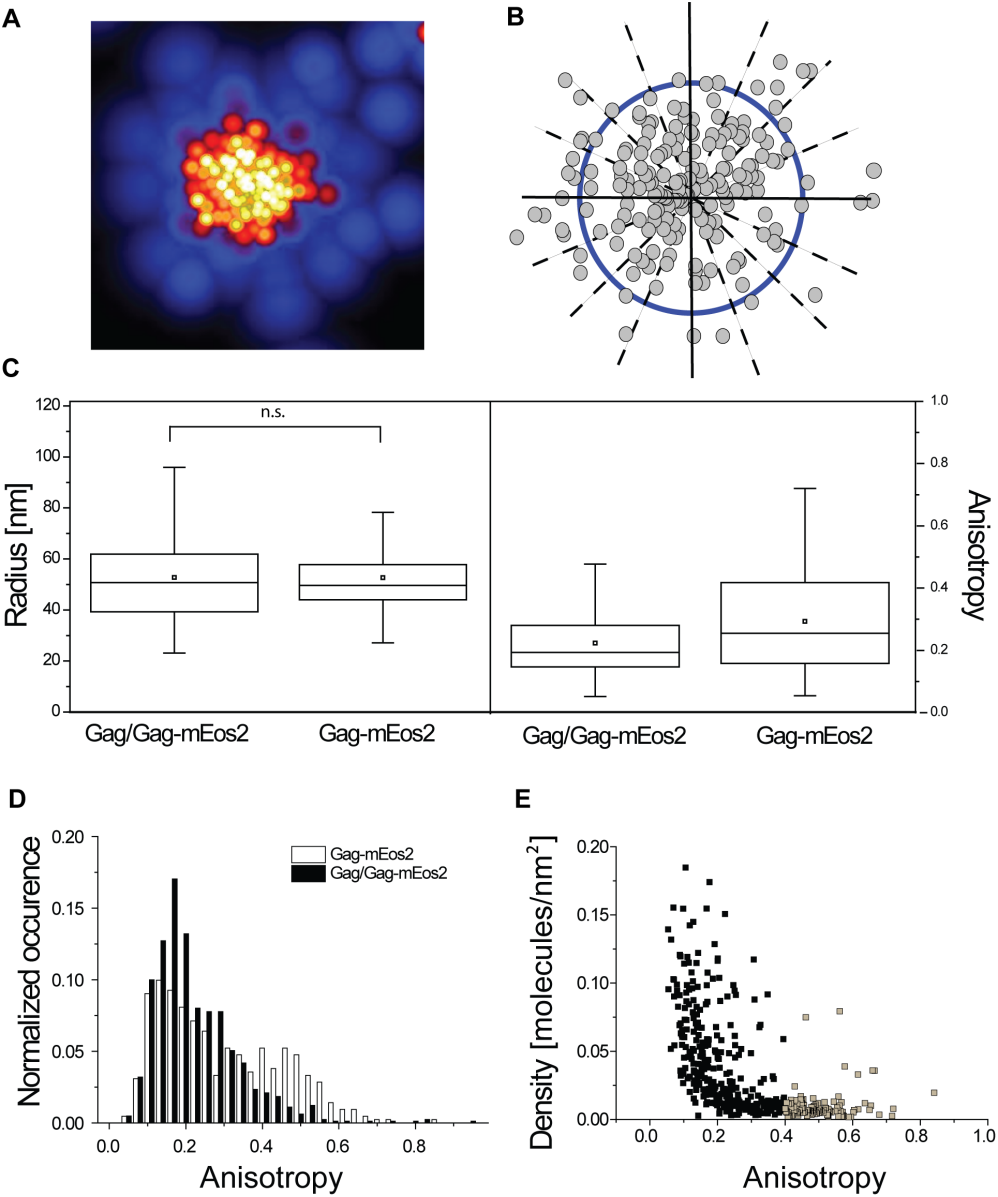
Quantification of the morphology of unlabeled and monomerically labeled Gag. (*A*) PALM image of VLP formed from Gag-mEos2 only. (*B*) Schematic illustration of computation of mean radius (blue circle) and radial anisotropy calculated from radial sectors (dashed lines). (*C*) Radius and radial anisotropy, defined as the coefficient of variation of the radius over all 16 sectors, for cells expressing Gag-mEos2 only or Gag and Gag-mEos2. Dots indicate mean, horizontal line within the box median, boxes indicate 25^th^ and 75^th^ percentiles and vertical bars 5^th^ and 95^th^ percentiles. (*D*) Normalized distribution of radial anisotropy (N>400). (*E*) Density of Gag clusters for cells expressing Gag-mEos2 only as a function of the radial anisotropy. Clusters with high anisotropies (gray squares) tend to display low densities.

To study the effect of Gag labeling on the morphology of membrane-bound VLPs in formation, we identified Gag clusters from the PALM images and extracted their morphological features by studying the distribution of molecules within 16 radial sectors from the cluster’s center of mass as previously described [7]. This approach permits the determination of the mean radius (Fig. 6, *A-C*) as well as the coefficient of variation of the radius (Fig. 6*C*) of each cluster. The radius provides a measure of the size of individual assembling VLPs, whereas the coefficient of variation of the radius provides a measure of their radial anisotropy. The radial anisotropy allows us to quantify how “disc-like” individual membrane-bound Gag clusters are. The more a given cluster differs form a symmetric round shape, the higher its radial anisotropy.

Interestingly, VLPs formed from Gag or Gag together with Gag-mEos2 show radii with no significant differences (Fig. 6*C*). This observation is independent of protein stoichiometry, as one would expect if there were no impact of the mEos2 label on the molecular packing of Gag. We measured average radii of 53±12 nm for Gag-mEos2 only and 53±17 nm for VLPs formed from Gag/Gag-mEos2. The radial anisotropy shows a broader distribution shifted to slightly higher values for Gag-mEos2 only (Fig. 6*C*). A closer analysis of the distribution of anisotropies revealed a double peak, accentuated in the case of Gag/Gag-mEos2 (Fig. 6*D*). The first peak represents clusters of Gag-mEos2 with nearly isotropic shapes, with a width that is due to the imprecise localization of molecules [7]. The second peak corresponds to Gag clusters having anisotropic morphologies and accounts for ∼25% of all clusters. In the case of co-expressed unlabeled and labeled Gag this second peak is reduced, comprising ∼9% of all clusters (Fig. 6*D*). We further analyzed the clusters with higher radial anisotropy, and found that the majority (90%) of Gag-mEos2 clusters belonging to this second peak fall within the category of low density clusters of less than 0.02 mol/nm^2^ (Fig. 6*E*). Thus, we surmise that these clusters correspond to preassembling or nucleating structures. Because of their low density, they would not be efficiently detected when unlabeled Gag is titrated in.

The results obtained by PALM combined with molecular counting and a quantitative fluorescent reporter for unlabeled Gag allowed us to directly show that monomerically labeled and mixtures of unlabeled and labeled Gag assemble into morphologically indistinguishable VLPs.

## Discussion

The primary requirement for the use of fluorescent labels is that they should not interfere with protein function or spatial organization. Tests of the functionality of a protein fusion are generally designed depending on the specific protein under study. In the case of Gag, its assembly into VLPs constitutes this functional test. Native spatial organization of the protein fusion at the microscale in turn can and should be tested by complementary immunostaining and standard fluorescence imaging [5]. Changes in the spatial organization at the nanoscale, however, are generally not queried because they are difficult to measure. These changes can be substantial, especially for densely packed proteins such as Gag [7], [8], therefore calling into question the biological relevance of observations using FP tags.

PALM imaging combined with molecular counting analysis provides a unique tool to extract nanoscale information on molecular packing and number of molecules per assembling membrane-bound cluster of Gag. We used this method to confirm that unlabeled and labeled Gag proteins are incorporated into assembling VLPs, by showing that the molecular density decreases when unlabeled Gag is co-expressed. Moreover, the superior resolution of this technique allowed us to show that membrane-bound Gag clusters formed from Gag-mEos2 and Gag/Gag-mEos2 are morphologically indistinguishable, an information until recently only accessible with electron microscopy techniques, which unfortunately lack protein specificity.

Interestingly, this is not true for the tandem-dimeric version of Eos (td-Eos) or other chimeric Gag fusions [29]. We previously showed that tagging Gag with tdEos (Gag-tdEos) leads to a nearly 2-fold increase in the size of membrane-bound Gag custers [7]. Using the same approach presented here for Gag-mEos2, we observed that the distribution of radii of membrane-bound Gag clusters shifts to a lower average for a mixture of Gag and Gag-tdEos as compared to Gag-tdEos only (see S6 Figure in S1 File), indicating that viral assembly is perturbed by the bigger tandem-dimeric label at the level of Gag assembly at the membrane. Thus, for specific labeling schemes the co-expression of helper Gag is not only necessary to rescue native infectivity, but also to rescue native morphology of membrane-bound, forming VLPs. For this co-expression to be consistent, we show that single cell measurements of VLP assembly require a quantitative read-out for the presence of unlabeled Gag. Furthermore, in transient co-transfection the simultaneous uptake and expression of both plasmids is not guaranteed at the single cell level, which enforces the need for such a read-out. As a consequence, the co-transfection ratio of unlabeled to labeled Gag plasmid of 10 to 20 fold that has been previously proposed [8] is only meaningful with such a quantitative read-out, given that the unlabeled to labeled Gag ratio can be subject to fluctuations over an order of magnitude.

## Conclusion

Using a fluorescent reporter protein for unlabeled Gag, we have shown that for the co-transfection rescue to be meaningful a quantitative read-out for the expression of unlabeled Gag is necessary. We have moreover shown by PALM imaging combined with molecular counting and quantitative image analysis that monomerically labeled and mixtures of unlabeled and labeled Gag assemble into morphologically indistinguishable clusters at the membrane. The quantitative measurements described here can be applied in general to determine the impact of protein modifications on nanoscale organization.

## Supporting Information

S1 File **Contains all supporting figures.** S1 Figure. Co-transfection of Gag-GFP and Gag-mCherry. Hela cells were transfected with Gag-mCherry only (left), Gag-GFP only (middle) or a mixture of both plasmids (right). Scale bars correspond to 200 µm. S2 Figure. Cell-to-cell variability in the co-expression levels of H2B-mPlum and Gag. Each symbol corresponds to a single cell. Immunostaining of Gag was performed as described in Materials and Methods. S3 Figure. FCS measurements of mEos2. (A) Schematic illustration of FCS calibration with mEos2 in solution (excited analyte). (B) Measured photon count rate for 120 nM recombinant mEos2 in solution. (C) Autocorrelation curve, fitted decay and residuals for the same data set. (D) Typical Gag-mEos2 low expressing cell chosen for the calibration of mEos2 concentration in cells. Scale bar: 10 µm (E) Measured photon count rate for cell shown in (D). (F) Autocorrelation curve, fitted decay and residuals for the same data set. S4 Figure. FCS measurements of mPlum. (A) Schematic illustration of FCS calibration with mPlum in solution (excited analyte). (B) Measured photon count rate for 350 nM recombinant mPlum in solution. (C) Autocorrelation curve, fitted decay and residuals for the same data set. (D) Typical Gag-mEos2 low expressing cell chosen for the calibration of mPlum concentration in cells. Scale bar: 10 µm (E) Measured photon count rate for cell shown in (D). (F) Autocorrelation curve, fitted decay and residuals for the same data set. S5 Figure. Calibration curves of recombinant mEos2 and mPlum in solution. (A) Concentration of mEos2 measured by absorption as a function of concentration measured with FCS. (B) Same data for mPlum. S6 Figure. Normalized size distribution of nascent VLPs for Gag-mEos2 and Gag-tdEos only and Gag-tdEos co-expressed with unlabeled Gag. Gag-tdEos forms biger VLPs as compared to Gag-mEos2. The size distribution for Gag-tdEos shifts to lower values when an unlabeled form of Gag is co-expressed.
(PDF)
