# Supplementary material for: A Quantitative Approach to Evaluate the Impact of Fluorescent Labeling on Membrane-Bound HIV-Gag Assembly by Titration of Unlabeled Proteins
Source: PLoS One. 2014 Dec 10;9(12):e115095. doi: 10.1371/journal.pone.0115095 (PMC4262470; doi:10.1371/journal.pone.0115095)
Supplement: S1 File — Contains all supporting figures. S1 Figure. Co-transfection of Gag-GFP and Gag-mCherry. Hela cells were transfected with Gag-mCherry only (left), Gag-GFP only (middle) or a mixture of both plasmids (right). Scale bars correspond to 200 µm. S2 Figure. Cell-to-cell variability in the co-expression levels of H2B-mPlum and Gag. Each symbol corresponds to a single cell. Immunostaining of Gag was performed as described in Materials and Methods. S3 Figure. FCS measurements of mEos2. (A) Schematic illustration of FCS calibration with mEos2 in solution (excited analyte). (B) Measured photon count rate for 120 nM recombinant mEos2 in solution. (C) Autocorrelation curve, fitted decay and residuals for the same data set. (D) Typical Gag-mEos2 low expressing cell chosen for the calibration of mEos2 concentration in cells. Scale bar: 10 µm (E) Measured photon count rate for cell shown in (D). (F) Autocorrelation curve, fitted decay and residuals for the same data set. S4 Figure. FCS measurements of mPlum. (A) Schematic illustration of FCS calibration with mPlum in solution (excited analyte). (B) Measured photon count rate for 350 nM recombinant mPlum in solution. (C) Autocorrelation curve, fitted decay and residuals for the same data set. (D) Typical Gag-mEos2 low expressing cell chosen for the calibration of mPlum concentration in cells. Scale bar: 10 µm (E) Measured photon count rate for cell shown in (D). (F) Autocorrelation curve, fitted decay and residuals for the same data set. S5 Figure. Calibration curves of recombinant mEos2 and mPlum in solution. (A) Concentration of mEos2 measured by absorption as a function of concentration measured with FCS. (B) Same data for mPlum. S6 Figure. Normalized size distribution of nascent VLPs for Gag-mEos2 and Gag-tdEos only and Gag-tdEos co-expressed with unlabeled Gag. Gag-tdEos forms biger VLPs as compared to Gag-mEos2. The size distribution for Gag-tdEos shifts to lower values when an unlabeled form of Gag is co-expressed. (PDF) [file pone.0115095.s001.pdf]

Supporting Information

A quantitative approach to evaluate the impact of fluorescent labeling on membrane-bound HIV-Gag assembly by titration of unlabeled proteins

Julia Gunzenhäuser[1], Romain Wyss[2] and Suliana Manley[1]

[1]Laboratory of Experimental Biophysics, Ecole Polytechnique Fédérale de Lausanne, Lausanne, Switzerland

[2]Laboratory of Physical Chemistry of Polymers and Membranes, Ecole Polytechnique Fédérale de Lausanne, Lausanne, Switzerland

FIGURE S1 Co-transfection of Gag-GFP and Gag-mCherry.

FIGURE S2 Cell-to-cell variability in the co-expression levels of Gag and H2B-mPlum for the single plasmid co-expression system and standard co-transfection.

FIGURE S3 FCS measurements of mEos2.

FIGURE S4 FCS measurements of mPlum.

FIGURE S5 Calibration curves of recombinant mEos2 and mPlum in solution.

FIGURE S6 Co-expressing Gag-tdEos together with unlabeled Gag partly rescues the observed increase in VLP size.

FIGURE S1

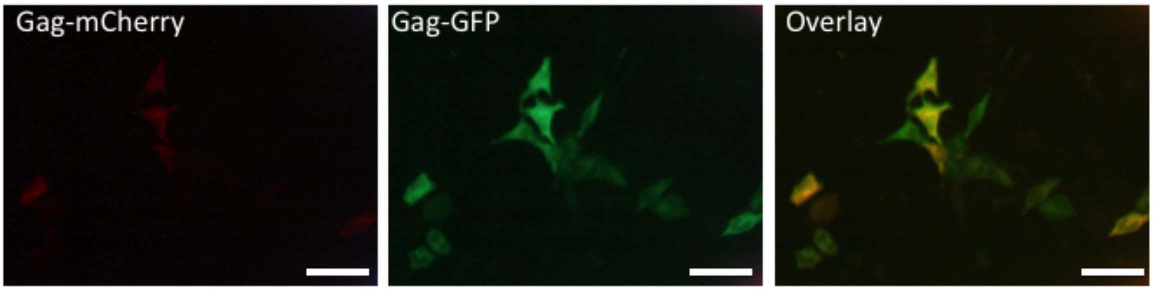

Hela cells were transfected with Gag-mCherry only (left), Gag-GFP only (middle) or a mixture of both plasmids (right). Scale bars correspond to 200 µm.

FIGURE S2

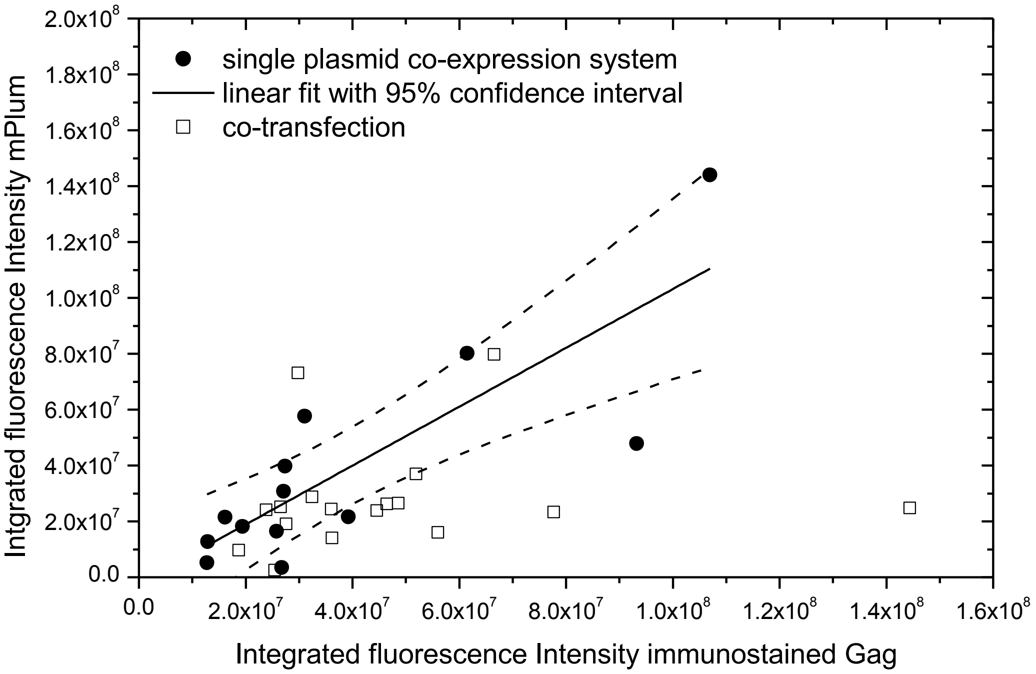

Cell-to-cell variability in the co-expression levels of H2B-mPlum and Gag. Each symbol corresponds to a single cell. Immunostaining of Gag was performed as described in Materials and Methods.

FIGURE S3

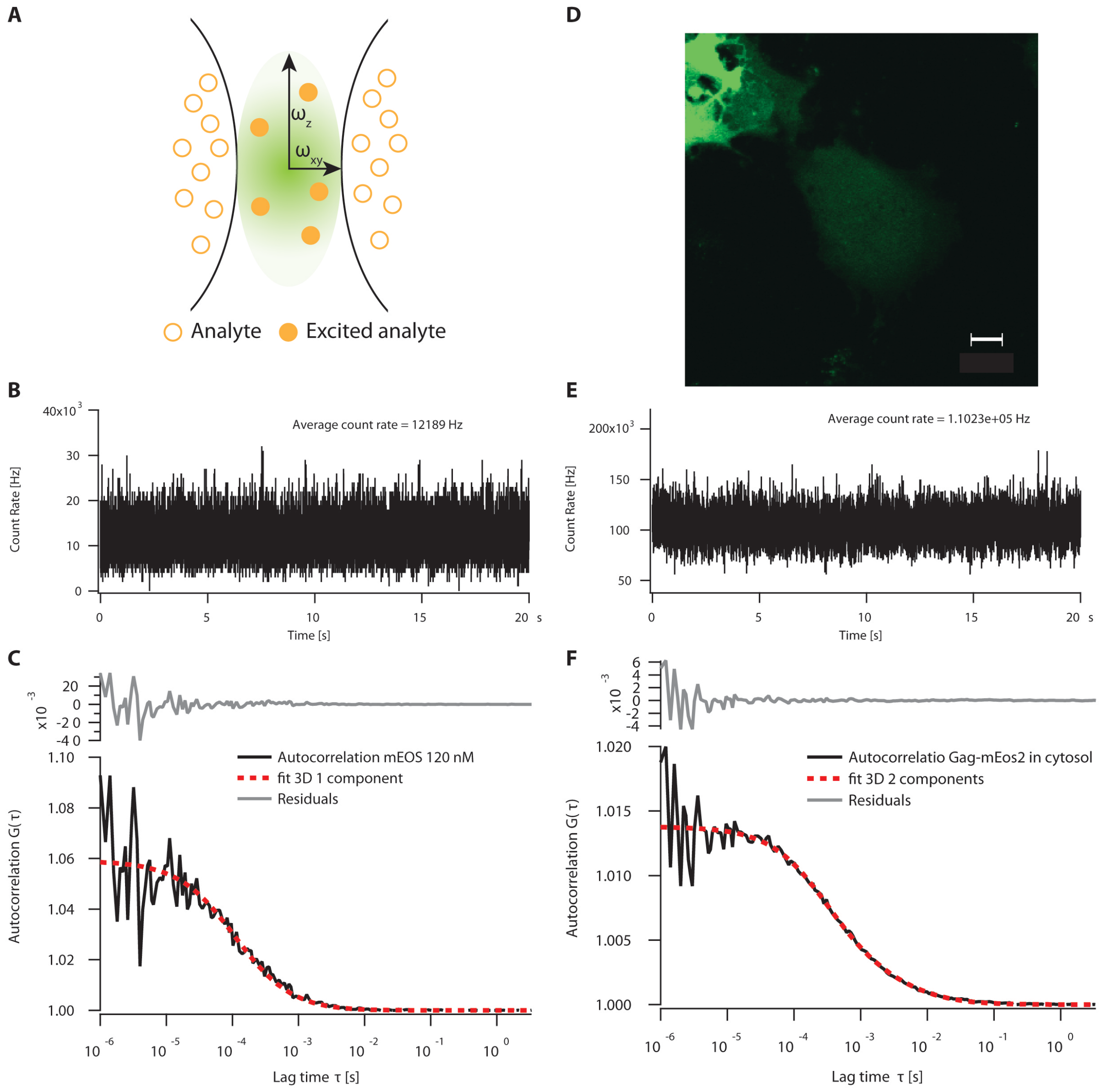

FCS measurements of mEos2. (A) Schematic illustration of FCS calibration with mEos2 in solution (excited analyte). (B) Measured photon count rate for 120 nM recombinant mEos2 in solution. (C) Autocorrelation curve, fitted decay and residuals for the same data set. (D) Typical Gag-mEos2 low expressing cell chosen for the calibration of mEos2 concentration in cells. Scale bar: 10 μm (E) Measured photon count rate for cell shown in (D). (F) Autocorrelation curve, fitted decay and residuals for the same data set.

FIGURE S4

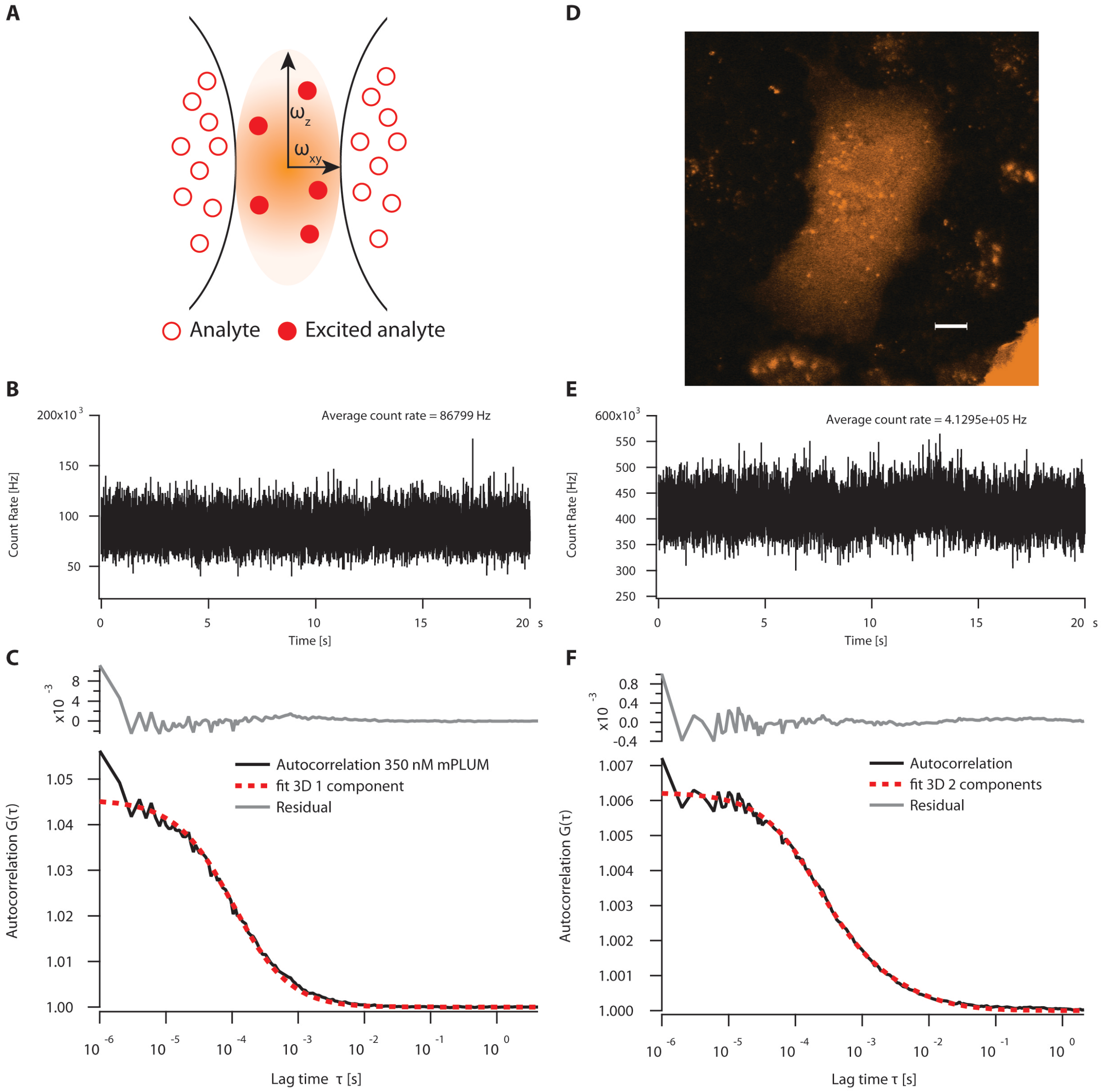

FCS measurements of mPlum. (A) Schematic illustration of FCS calibration with mPlum in solution (excited analyte). (B) Measured photon count rate for 350 nM recombinant mPlum in solution. (C) Autocorrelation curve, fitted decay and residuals for the same data set. (D) Typical Gag-mEos2 low expressing cell chosen for the calibration of mPlum concentration in cells. Scale bar: 10 μm (E) Measured photon count rate for cell shown in (D). (F) Autocorrelation curve, fitted decay and residuals for the same data set.

FIGURE S5

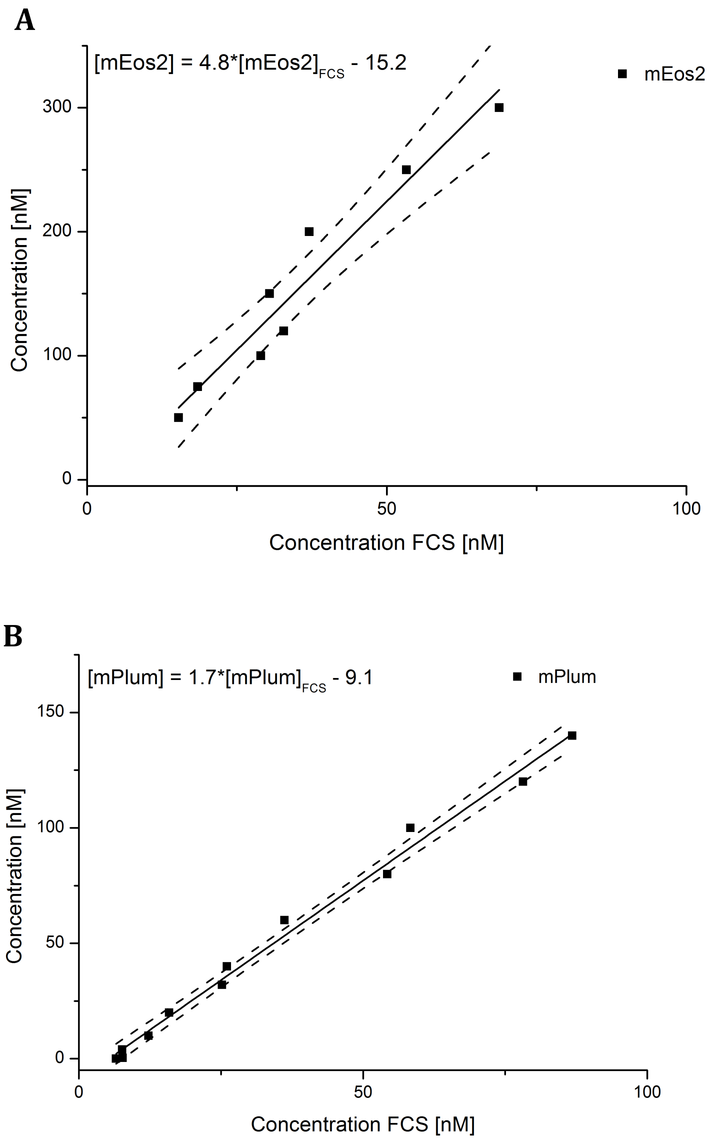

Calibration curves of recombinant mEos2 and mPlum in solution. (A) Concentration of mEos2 measured by absorption as a function of concentration measured with FCS. (B) Same data for mPlum.

FIGURE S6

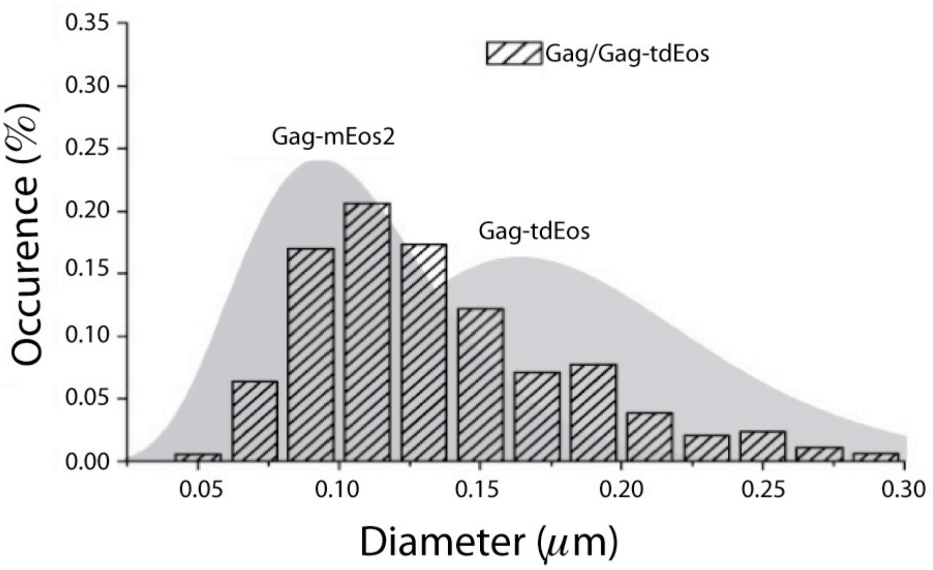

Normalized size distribution of nascent VLPs for Gag-mEos2 and Gag-tdEos only and Gag-tdEos co-expressed with unlabeled Gag. Gag-tdEos forms biger VLPs as compared to Gag-mEos2. The size distribution for Gag-tdEos shifts to lower values when an unlabeled form of Gag is co-expressed.
